# Service readiness of Public and private health facilities for roll-out of hepatitis C screening and management: a cross-sectional study

**DOI:** 10.1186/s12913-025-13684-8

**Published:** 2026-02-20

**Authors:** Asma Ummad, Amna Rehana Siddiqui, Aroosa Nighat, Atif Riaz, Hasan Nawaz Tahir, Saeed Hamid, Shehla Zaidi

**Affiliations:** 1 APPNA Institute of Public Health, Jinnah Sindh Medical University, Karachi, Pakistan; 2 The Aga Khan University, Karachi, Pakistan; 3 Department of Community Health Sciences, The Aga Khan University, Karachi, Pakistan

**Keywords:** Hepatitis c, Service readiness, Direct-acting antivirals, Public–private mix, Pakistan

## Abstract

**Background:**

Direct-acting antiviral (DAA) therapy has transformed hepatitis C management by enabling cost-effective outpatient treatment. Pakistan, with one of the world’s highest hepatitis C burdens, has set ambitious policy targets for the rollout of DAA therapy supported by rapid screening and confirmatory testing. This study assessed the service readiness of public and private healthcare facilities to inform future scale-up of hepatitis C services in Pakistan.

**Methods:**

Between June 2021 and January 2022, a purposive sample of 39 facilities was selected from 14 high-burden districts in consultation with the National Hepatitis Program: 15 public hospitals, 15 private hospitals, 6 standalone private laboratories, and 3 private blood banks. A cross-sectional descriptive assessment was conducted to evaluate service availability, commodity stocks, staff training, quality-of-care guidelines, reporting mechanisms, supervision, and community engagement. Data were collected through structured questionnaires administered to facility managers and staff, supplemented by an observation checklist.

**Results:**

DAA therapy was available at 10/15 (66.7%) public hospitals and 13/15 (86.7%) private hospitals. HCV screening was offered at 9/15 (60.0%) public hospitals compared to 3/15 (20.0%) private hospitals, with most private hospitals referring patients to standalone laboratories. Case notification was reported by 6/15 (40.0%) public facilities and 4/15 (26.7%) private facilities. In the public sector, DAA stock was available at 7/15 (46.7%) hospitals, but 5/10 (50.0%) of those offering treatment reported recent stock-outs. Private facilities generally prescribed DAAs from external pharmacies. Private laboratories had stronger systems for equipment calibration (4/5, 80.0%) and electronic reporting (5/6, 83.3%), while public facilities were more often supported with standardized guidelines (10/15, 66.7%). Awareness of community referral campaigns remained low in both sectors (≈20–25%).

**Conclusion:**

This study found that public facilities were stronger in programmatic guidance, training, and supervision, whereas private facilities demonstrated greater functionality in equipment maintenance and electronic reporting. However, both sectors faced significant gaps: public facilities experienced resource shortages and relied heavily on paper-based systems, while private facilities lacked integration with case notification and standardized guidelines. Scaling up the provision of HCV testing and DAA therapy in Pakistan and similar LMICs requires quality leadership and electronically supported information systems to precede future extensive investment in commodities. Leveraging the comparative strengths of private facilities, services, resources, and online reporting, alongside strengthening public supply chains, will be critical to achieving universal access to hepatitis control services.

## Introduction

Hepatitis C Virus (HCV) infection is bloodborne and can lead to liver cirrhosis and cancer (WHO, 2016). The advent of direct-acting antiviral (DAA) therapies has revolutionized HCV treatment, achieving sustained virologic response rates exceeding 90% across all genotypes and in special populations, including those with HIV/HCV coinfection [1]. Pakistan has the second-largest HCV burden worldwide after Egypt, with an estimated 8 million infections [2].

Egypt’s incorporation of DAA therapy and a targeted screening strategy for outpatient treatment referrals has been a success story for deliberative policy interventions in hepatitis C control [3]. Similar approaches have been adopted in India, where the National Viral Hepatitis Control Program offers free screening and treatment using generic DAAs [4], and in Georgia, which provides free testing and treatment through a nationally coordinated hepatitis C elimination program [5].

The National Hepatitis Survey conducted in Pakistan between 2007 and 2008 reported an overall prevalence of HCV of 4.9%, attributed to increasing age and medical care-related practices [6]. Subsequent surveys estimated an HCV infection rate of 6.2% in the general population of Pakistan [7]. Access to care, surveillance, diagnosis, and treatment rates are disproportionately low in LMICs, including Pakistan, as compared to the actual burden of HCV [8]. Despite the development of HCV elimination targets by the World Health Organization, a gap remains in achieving the intended results [9]. To address this concern, a Global Investment Framework was developed, which helped countries identify funding sources and provided them with methods to rationalize investments in HCV elimination efforts economically [9].

The Hepatitis Prevention and Control Program (HPCP) has been operational in Pakistan since 2005, spanning both federal and provincial levels through joint activities and co-financing (Asian Development Bank [10]). The HPCP has been supporting public sector secondary care facilities for diagnosis and treatment, with the establishment of focal public sector sites in each district. There are also occasional community-based campaigns for building household awareness and encouraging referrals to focal facilities.

In response to the WHO Global Health Sector Strategy (GHSS) for the elimination of HCV infections, Pakistan developed the National Hepatitis Strategic Framework (NHSF) 2017–2021, outlining a roadmap for the elimination of HCV infections by 2030 [11]. A primary objective of the NHSF is to rapidly scale up screening, testing, and DAA therapy, supported by strengthening health information systems, supply chain management, and improving risk communication for timely screening. Hence, rapid screening, followed by PCR-based testing and provision of DAA therapy, is proposed as a game-changer to be implemented, relying on sufficient capacity at healthcare facilities to progressively reach an estimated 888,000 cases per year, in line with the disease burden modelling provided [12].

Although the HPCP support has been confined to public sector facilities, the policy intent under the NHSF is to include private sector facilities within Pakistan’s mixed healthcare system as part of the DAA roll-out, maximizing reach to potential HCV cases.

A multi-faceted exploratory gap analysis study of the Hepatitis C program in Pakistan was commissioned by the government to inform the implementation of the proposed program and guide the systematic development of a range of programmatic capacity-building and investment options [13]. This paper draws on service delivery assessment data from the larger study, with the objective of providing evidence on the service readiness of public and private healthcare facilities in selected HCV-endemic districts of Pakistan for scaling up HCV rapid screening, PCR testing, and DAA treatment to achieve elimination targets. It assesses the existing status in terms of the availability of services, resource availability for service provision, measures for technical quality, information reporting, supervision support, and links with community engagement activities. A deliberate focus was placed on assessing private facilities alongside programmatically supported public sector facilities to help inform private sector engagement options for supporting service delivery. The findings are intended for policymakers, practitioners, and healthcare researchers to inform the expansion of cost-effective hepatitis C treatment, as well as to provide a tracer lens for facility readiness assessments among health systems researchers.

## Method

### Research design

We conducted a cross-sectional facility-based survey between June 2021 and January 2022 to examine the readiness of public and private healthcare facilities for hepatitis C screening, confirmatory PCR testing, and DAA therapy. This rapid facility assessment was conducted as part of a larger, multifaceted gap analysis of the National Hepatitis C Program (NHCP), commissioned by the Asian Development Bank to inform national-scale planning. The survey employed a case study design, focusing on programmatically relevant indicators that could be collected practically across both public and private health facilities. For clarity, we define several key terms: case notification refers to the mandatory reporting of confirmed HCV-positive cases to public health authorities through either paper-based or electronic systems; patient data recording refers to maintaining patient demographic, diagnostic, and treatment records at the facility level (distinct from a formal disease registry); and reporting links refer to the communication and data-sharing channels between facilities and higher-level public health or administrative agencies for program monitoring and surveillance.”

## Study settings

Pakistan comprises four provinces, which are further administratively divided into districts with healthcare organized under a district health system. There is a total of 154 districts across the four provinces, as well as the federal district of Islamabad City [14]. Healthcare service delivery is decentralized, with hepatitis services overseen by provincial health departments and directly by the federal health ministry in Islamabad city district [15–19].

Resourcing for hepatitis C control and treatment services has been historically inadequate but is set to be boosted with support from the government and development partners under current planning. The private health sector is the frontline major provider of ambulatory care, diagnostic services, and blood transfusion services in Pakistan and has grown over the years due to weaknesses in the public sector health system (WHO, 2016). The private sector relies heavily on out-of-pocket payments by patients, which limits affordable access to hepatitis and other healthcare services. Private laboratories, blood banks, and outpatient facilities have not yet received support from the Hepatitis Prevention and Control Program (HPCP), which has thus far been focused on public sector facilities.

## Sampling

Given the case study approach applied in the assessment, a sample of fourteen districts with a high caseload of Hepatitis C was purposively selected for service assessment in consultation with the provincial and federal Hepatitis C programs. For geographic representation, the capital city district of Islamabad, four districts from each of the three most populous provinces (Punjab, Sindh, and Khyber Pakhtunkhwa), and one from the least populous province (Balochistan) were selected in consultation with the HPCP. The choice of districts by HPCP was based on high case reporting of Hepatitis C as well as a balance between urban and rural districts.

The District Health Offices facilitated access to both public and private sector health facilities. Two facilities, including one private and one public facility, were selected per district for service assessment. The public sector focal facility for Hepatitis C treatment and testing within each district was identified for inclusion by provincial and federal HPCPs and comprised of secondary government hospitals equipped with laboratory services. A total of 15 public sector secondary hospitals were included, one from each district (Table 1). Matching private secondary hospitals were selected in each district, having similar bed capacities and being registered with the healthcare commission, as identified by the district health offices. In total, 15 private secondary hospitals, one in each district, were purposively selected (Table 1). Since private secondary facilities typically do not have in-house diagnostic services, patients were also referred to standalone private laboratories, in addition to sampling six private laboratories and three private blood banks. In total, 39 service delivery facilities were included across the four provinces and the Islamabad Capital Territory (ICT) (Table 1).

**Table 1:** Participating public & private facilities by province and district

Location District (Province)	Public facilities	Private Facilities	
	Secondary Care Facilities	Secondary Care Facilities	Standalone laboratories/blood banks
**Islamabad (Capital city)**	-	1	6 private laboratories 3 private blood banks
**Hafizabad (Punjab)**	1	1	
**Nankana Sahib (Punjab)**	2	1	
**Sialkot (Punjab)**	1	1	
**Vehari (Punjab)**	1	3	
**Karachi Central (Sindh)**	2	2	
**Karachi Malir (Sindh)**	1	-	
**Khairpur (Sindh)**	1	1	
**Sanghar (Sindh)**	1	-	
**Peshawar (Khyber-Pakhtunkhwa)**	1	1	
**Kohat (Khyber-Pakhtunkhwa)**	1	1	
**Batkhela (Khyber-Pakhtunkhwa)**	1	1	
**Haripur (Khyber-Pakhtunkhwa)**	1	1	
**District Quetta (Balochistan)**	1	1	
**Total**	**15**	**15**	**9**

## Data tools and collection

Information was collected through semi-structured questionnaires and an observation checklist. The questionnaire was administered to medical superintendents and the facility in charge, as well as to medical doctors and laboratory technicians, and collected information on services provided, training, guidelines, reporting links, supervisory visits, and awareness of community activities (Box 1). An observation checklist was used to assess supply stocks, availability of information technology, and guiding manuals (Box 1). The tools drew on thematic categories from the World Health Organization’s Service Availability and Readiness Assessment (SARA) tool [20]. Still, they were simplified and adapted in line with programmatic indicators of the HPCP, the time constraints of a rapid assessment, and the practicalities of accessing data from private providers. Staff knowledge assessments and direct observations of the service delivery process were not conducted. Information was collected on i) supplies availability, ii) human resource availability, iii) case screening, iv) technical guidance and training, v) monitoring and reporting systems, vi) supervision, and vii) links with community awareness programs. The tools were reviewed by NHCP and finalized with field testing in one district to ensure relevance, clarity, and feasibility in the local context. Feedback from data collectors and facility staff during the field testing informed minor modifications to align the tools with regional perspectives and ensure cultural appropriateness.

Data was collected by two trained team members, supervised by a public health epidemiologist, and followed by data review with the study team lead. Training sessions were conducted for the two data collectors to ensure they understood the administration of the tool, conducting interviews, and observations. Data was collected with written informed consent, and personal identifiers were anonymized for analysis. Data was entered in real-time electronically using Google Forms, which included built-in validation commands to minimize errors. Post-data collection meetings were held regularly to ensure quality checks, facilitating the resolution of data inconsistencies and gaps. The systematic and iterative process, along with structured quality assurance measures, ensured the reliability and validity of the data collected for the study.

**Box 1:** Study category, areas, and data sources

Data Categories	Areas	Data Source
**Facilities**		
Services available	• HCV screening test (Rapid Detection Test), HCV Confirmatory Test (PCR Test) • DAA therapy	Questionnaire
Resource availability	• Stocks of Tablet Sofosbuvir and Tablet Daclatasvir • Availability of internet, telephone, and mobile phone	Observation
Recording and reporting	• Patient data recording • Reporting links, reporting format	Observation Questionnaire
Training System	• Ever received training, source of training • Guidelines received to manage HCV patients	Questionnaire Observation
Quality of care guidelines	• Types of guidelines in place • Type of algorithm used	Observation Questionnaire
Supervision	• Monitoring or supervisory visits	Questionnaire
Community Engagement	• Community campaign to refer patients • Other community engagement initiatives	Questionnaire
**Laboratory**		
Services available	• Rapid and Elisa test • Other test performed on HCV patients	Questionnaire
Recording and reporting	• Patient data recording • Reporting links, reporting format	Observation Questionnaire
Training System	• HCV training received by laboratory staff	
Resource Availability	• Availability of lab reagents	Observation
	• Service standards for equipment periodic calibration	Observation
**Blood Banks**		
Quality of care practices	• Staff vaccinated for HBV	Questionnaire
	• Staff screened for HCV	Questionnaire
Recording and reporting	• Patient data recording • Reporting links, reporting format	Observation Questionnaire

## Results

Overall, the survey of 39 facilities revealed complementary strengths and weaknesses across sectors. Public hospitals more often had programmatic guidance, trained staff, and supervision, whereas private facilities had stronger equipment functionality and electronic reporting systems. Stock-outs and reliance on paper-based reporting were common in the public sector, whereas private facilities often lacked integration with case notification and programmatic guidelines. Community engagement remained weak across both sectors

## Public and private secondary healthcare facilities

### Service availability

DAA therapy was available at 10/15 (66.7%) public hospitals and 13/15 (86.7%) private hospitals. Screening was offered at 9/15 (60.0%) public hospitals compared to 3/15 (20.0%) private hospitals, with most private hospitals referring patients to standalone laboratories. Case notification was reported by 6/15 (40.0%) public and 4/15 (26.7%) private facilities (Table 2).

**Table 2:** Functional services and resources at public and private secondary facilities, service availability – screening, testing, daa therapy

	Public facilities	Private facilities
**HCV screening based on ELISA rapid test) (last month)**	60%	20% Refer to standalone private laboratories
**HCV PCR testing (last month)**	-	Refer to standalone private laboratories
**Case notification**	40%	25%
**DAA therapy provided to HCV patients (last month)**	66%	90%
**Resources availability – DAA stocks, trained staff, reporting resources**		
	Public facilities	Private facilities
**Availability of DAA**		
**Tablet Sofosbuvir**	46%	NA
**Tablet Daclatasvir**	46%	NA
**Stock out of DAA amongst those offering treatment (during last 3 months)**	50%	NA
**Staff standalone training in HCV diagnosis/management/drug dispensation from any source (ever received)**		
**General Doctors**	66%	60%
**Specialist doctors**	60%	53%
**Laboratory Technician**	40%	NA
**Pharmacy**	33%	NA
**Resources for reporting:**		
**Availability of Telephone**	66%	80%
**Availability of Internet**	66%	66%
**Availability of Mobile phones**	66%	66%

## Resource availability

DAA stocks were available at 7/15 (46.7%) public hospitals, but 5/10 (50.0%) of those offering treatment reported stock-outs in the preceding three months. Private hospitals prescribed DAAs from external pharmacies rather than maintaining in-house stock. Training was reported by 66% of general doctors and 60% of specialist doctors in public hospitals, while no pharmacists or laboratory technicians in private facilities reported training. Access to telephone lines was higher in private hospitals (80%) than public ones (66%), although both sectors reported similar internet and mobile phone access (Table 2).

## Recording and reporting

In public facilities, 50% recorded data for positive patients, 45% registered follow-up, and 40% submitted case notifications to District Health Offices. In private hospitals, 60% recorded positive cases, but 25% registered follow-up and 10% reported cured cases. Case notification was reported by 25% of private hospitals, typically through electronic systems (Table 3).

**Table 3:** Disease reporting, quality assurance, supervision, community links, disease reporting and quality assurance

	Public	Private
**Patient data recorded for those tested/screened +ve (last month)**	50%	60%
**Patient data registered for follow up (last month)**	50%	25%
**Data entered for HCV patients declared cured (last month)**	45%	10%
**Case notification report is submitted by the facility**	40%	25%
**Mechanism of disease notification**	Report to DHO	Electronic when asked
**Case reporting system in place for other diseases**	TB, HIV, NGO	TB, Polio, HIV
**Algorithm used by MO for HCV infection management**		
**Provided by Hepatitis Program**	66%	25%
**Other/self**	34%	75%
**Received guidelines to manage HCV patients**	45%	10%
**Type of guideline received**	HPCP Booklet, Training on Treatment*, Guidelines 2020**, Standard Operating Procedure***	-
**Refresher training/sessions received by the facility in (last 1 year)**	40%	-
**Supervisory visits for HCV services**		
**Supervisory visit of HCV treatment/screening/testing by Hepatitis Programme (received during the last year)**	46%	-
**Supervisory visit of HCV treatment/screening/testing by District Health Office (received during the last year)**	53%	-
**Supervisory visit of HCV treatment/screening/testing by Senior Hospital Management (received during the last year)**	46%	30%
**Changes after supervision**	Waiting areas Infection control renovation	Separate room For HCV
**Links to community HCV activities**		
**Aware of community campaign for referring patients**	20%	25%
**Aware of other community awareness activities to refer patients**	20%	20%

*Sindh Government training. ** Punjab HCV program guidelines. ***ICP=Infection control program

## Guidelines

Program algorithms were available in 10/15 (66.7%) public hospitals, compared to 4/15 (25.0%) private hospitals. Formal guidelines were reported in 7/15 (45.0%) public facilities versus 2/15 (10.0%) private facilities. No private facilities reported participating in training, while 6/15 (40.0%) public hospitals reported receiving refresher training in the past year (Table 3).

## Supervision

Supervisory visits were reported by 7/15 (46.7%) public hospitals from the Hepatitis Program, 8/15 (53.3%) from District Health Offices, and 7/15 (46.7%) from senior hospital management. In the private sector, 30% of facilities reported visits from senior hospital management; no facilities reported visits from the Hepatitis Program or the District Health Office (Table 3).

## Community engagement

Awareness of community-based HCV referral campaigns was reported by 3/15 (20.0%) public and 4/15 (26.7%) private hospitals. Awareness of other referral activities was also low in both sectors (20.0%) (Table 3).

## Laboratory services

### Service availability

HCV screening training had been received by 8/15 (53.3%) public laboratories and 2/6 (33.3%) private laboratories in the past 10 years. Among public labs, 46% used rapid diagnostic tests and 8% used ELISA, while all private labs used rapid diagnostic tests and 15% also used ELISA.

## Service quality

Standards for equipment calibration were in place in 20% of public laboratories versus 80% of private laboratories. Stock-outs of diagnostic reagents were reported by 20% of public labs, but none of the private labs.

## Recording and reporting

Case notification was reported by 8/15 (53.3%) public and 2/6 (33.3%) private laboratories. While 83% of private labs had electronic reporting systems, only 20% of public labs did so, with most relying on registers (Table 4).

**Table 4:** HCV testing and reporting in laboratories

Services	Public Facility	Private Facility
**HCV screening training received by laboratory staff (last 10 years)**	53%	33%
**Type of test being used to diagnose HCV cases**	ICT, ELISA, RAPID test Immuno-chromatography	ELISA, Rapid test, others Haem/biochemical analyser
**Tests for screening:**		
**Rapid Test**	46%	100%
**Other-Elisa**	8%	15%
**Stockout of lab reagent in last three months**	20%	None
**Service standards for equipment periodic calibration are in place (any source)**	20%	80%
**Other tests performed on HCV +ve patients:**		
**HBsAg**	53%	100%
**HIV antibody**	53%	66%
**Blood sugar**	25%	66%
**Creatinine**	20%	-
**Recording and reporting systems**		
**Electronic information system**	20%	83%
**Printed Register information system**	53%	16%
**Case notification provided to government**	53%	33%

## Blood banks

### Service availability and quality

All public and private blood banks screened blood products for HCV. All public blood bank staff were vaccinated for HBV and screened for HCV, compared with 65% of staff in private blood banks who were vaccinated for HBV and 100% who were screened for HCV. Systems for mandatory reporting of needle stick injuries were in place at 44% of public and 65% of private blood banks.

## Recording and reporting

Case notification was reported by 65% of both public and private blood banks. Electronic reporting was used in all private blood banks (100%) but in none of the public blood banks. Reporting linkages with other communicable disease programs were reported by 20% of public and 33% of private blood banks (Table 5).

**Table 5:** HCV testing and reporting in blood banks

	Public Facility	Private Facility
**All staff handling blood products is vaccinated for HBV**	100%	65%
**All staff handling blood products if screened for HCV**	100%	100%
**Mandatory reporting of needle stick injuries**	44%	65%
**HCV reporting provided to higher authorities**	65%	65%
**Electronic reporting of HCV cases**	0	100%
**Reporting links for HIV, HCV/HBV, other programmes**	20%	33%

## Discussion

This study identified four key findings. First, public hospitals were more likely to have programmatic guidance, trained staff, and supervisory support. Second, private facilities demonstrated greater functionality in terms of equipment maintenance and electronic reporting. Third, both sectors faced significant weaknesses, including stock-outs and reliance on paper-based systems in public facilities, as well as limited integration with case notification and programmatic guidelines in private facilities. Finally, community engagement remained weak across both sectors, representing a missed opportunity for improved referral and case detection.

Public sector facilities, despite programmatic support, were affected by shortages of medicines and laboratory reagents, reflecting wider supply chain constraints reported across low- and middle-income countries (LMICs). The presence of trained staff and standardized guidelines in public facilities highlights the effectiveness of program inputs but also underscores the need for consistent resourcing to maintain continuity of care. Similar findings of strong technical guidance but recurring stock-outs have been documented in LMIC health programs, where medicine shortages disrupt otherwise well-structured services.

In contrast, private facilities provided DAA consultations, maintained stronger infrastructure, and demonstrated a wider adoption of electronic reporting; however, they lacked integration into programmatic systems and case notification frameworks. This mirrors findings from comparative reviews of public and private health systems, where private providers often show better service functionality but weaker linkages with national health governance structures [21, 22]. Experiences from Egypt, India, and Georgia illustrate how these gaps can be addressed: Egypt’s *100 Million Healthy Lives* campaign successfully leveraged public-private partnerships to expand testing and treatment, India’s National Viral Hepatitis Program introduced free DAAs supported by generic production, and Georgia adopted centralized governance and standardized protocols to ensure equitable care.

Globally, progress towards hepatitis C elimination is increasingly framed within the broader agenda of universal health coverage (UHC). Achieving elimination requires equitable access to essential services such as screening, confirmatory testing, and affordable DAAs, without imposing financial hardship. Strengthening both public and private sectors, and embedding hepatitis C programs into UHC frameworks, is critical for ensuring sustainability (WHO, 2016 [8]). Pakistan’s mixed health system provides an opportunity to harness private-sector strengths while addressing public-sector resource gaps to advance both hepatitis elimination and UHC commitments.

Moving forward, integration across public and private facilities, interoperable digital reporting systems, and sustainable domestic financing will be central to success. Countries such as Brazil have emphasized domestic production of generic DAAs, while others have negotiated reduced patent pricing to expand access. [23]. Lessons from the COVID-19 pandemic, where digital reporting systems enabled real-time coordination and resource allocation, can be applied to hepatitis C programs to improve efficiency and surveillance. Furthermore, integration with other disease programs such as HIV and blood safety initiatives can enhance resource optimization and improve monitoring of co-morbidities [24].

## Limitations

This study has several limitations. The purposive selection of high-burden districts and HPCP-designated facilities may limit the generalizability of findings. Private facilities were restricted to those registered with the healthcare commission and willing to participate, which may not represent the full spectrum of private providers. Matching facilities by bed size and service type across districts was not always feasible, which could introduce imbalance. Finally, staff competency assessments and direct observation of service delivery were not conducted, limiting the assessment of clinical quality. These considerations should be taken into account when interpreting the findings.

## Conclusion

Private facilities, although overlooked by the national program, offer comparative advantages in services, resources, and online reporting systems, which can be leveraged to expand universal access to affordable DAA therapy, supported by rapid screening and testing in Pakistan. Harnessing of private facilities will require electronically supported information systems and quality oversight, in addition to the traditional emphasis on commodities.

## Data Availability

Not applicable

## Abbreviations

COVID-19: Coronavirus Disease
DAA: Direct Acting Agent
GHSS: Global Health Sector Strategy
HBV: Hepatitis B Virus
HCV: Hepatitis C Virus
HPCP: Hepatitis Prevention and Control Program
ICT: Islamabad Capital Territory
MIS: Management Information System
PCR: Polymerase Chain Reaction
RDT: Rapid Detection Test
SoP: Standard Operating Procedure
